# Diagnostic Devices for Isothermal Nucleic Acid Amplification

**DOI:** 10.3390/s120608319

**Published:** 2012-06-14

**Authors:** Chia-Chen Chang, Chien-Cheng Chen, Shih-Chung Wei, Hui-Hsin Lu, Yang-Hung Liang, Chii-Wann Lin

**Affiliations:** 1 Institute of Biomedical Engineering, National Taiwan University, Taipei 10617, Taiwan; E-Mails: ccchang.ibme@gmail.com (C.-C.C.); braddomkimo@yahoo.com.tw (C.-C.C.); comet.comet@gmail.com (H.-H.L.); d94548002@ntu.edu.tw (Y.-H.L.); 2 Institute of Biomedical Electronic and Bioinformatics, National Taiwan University, Taipei 10617, Taiwan; E-Mail: makeabbw@gmail.com; 3 Center for Emerging Material and Advanced Devices, National Taiwan University, Taipei 10617, Taiwan

**Keywords:** isothermal nucleic acid amplification, NASBA, SDA, RCA, LAMP, HDA

## Abstract

Since the development of the polymerase chain reaction (PCR) technique, genomic information has been retrievable from lesser amounts of DNA than previously possible. PCR-based amplifications require high-precision instruments to perform temperature cycling reactions; further, they are cumbersome for routine clinical use. However, the use of isothermal approaches can eliminate many complications associated with thermocycling. The application of diagnostic devices for isothermal DNA amplification has recently been studied extensively. In this paper, we describe the basic concepts of several isothermal amplification approaches and review recent progress in diagnostic device development.

## 1. Introduction

Nucleic acid amplification is one of the most valuable tools in nucleic acid detection because it can amplify fewer than 10 target copies, significantly improving assay sensitivity. The polymerase chain reaction (PCR) was introduced by Mullis [1] and has since become an indispensable tool in numerous molecular research and diagnostic applications. Related advanced technologies, such as multiplex PCR, nested PCR, real-time PCR, and reverse transcription PCR (RT-PCR), have been used for bimolecular analysis. However, there are numerous features confining the applicability of PCR. The approach requires thermal cycling instrumentation, considerable expertise, and a substantial amount of space in routine diagnostic laboratories, thus limiting its use to highly sophisticated facilities. These limitations in current PCR-based techniques have spurred the development of a new molecular-biological technique known as isothermal nucleic acid amplification. The major difference between PCR and isothermal amplification are the temperature reaction condition requirements. Stringent reaction conditions, including thermal cycling steps at specific temperatures, are employed in PCR, whereas only a single optimal reaction temperature is required for the entire isothermal amplification reaction, thus providing simpler and more effective reaction conditions without expensive equipment. Additionally, isothermal DNA amplification produces longer DNA fragments than the conventional PCR technique. Overall, isothermal nucleic acid amplifications have greater amplification efficiency and produce higher DNA yields than PCR owing to their undisrupted and sustained enzyme activity.

With the advent of microfabrication technology, one of the directions taken to address the future needs of bioanalysis and clinical diagnosis is the development of micro total analysis systems (μTAS) or labs-on-a-chip (LOC). This scaling down capability supports an exceptional ability to miniaturize various functional units such as pumps and reactors, making it possible to integrate and automate processes into a microsystem. Additionally, it offers important advantages over bulk or large-scale analysis including rapid assay results, high-throughput screening, and low consumption of reagents. Further, the energy required for microfabrication and operation is remarkably reduced. Most importantly, these benefits make microchip systems amenable to near-patient and point-of-care testing. The development of DNA amplification microinstruments began in the 1990s, when the concepts of integrated microfluidic devices were introduced to take advantage of microfabrication technology for biological and chemical analyses [2]. To establish such a system, it was desirable to create a totally integrated device performing a series of specific molecular functions such as nucleic acid extraction and purification, nucleic acid amplification and detection, and other supporting analysis techniques, with minimal dead volumes.

Owing to the overwhelming quantity of literature available on isothermal DNA amplification devices, we will describe the strategies of five major isothermal techniques. Because several reviews have previously focused on isothermal methods in bioanalysis applications [3–5], we focus mainly on recent advances in the rational design and fabrication of integrated DNA microchips. The measurements of amplified DNA using different approaches will also be reviewed. Finally, future challenges and perspectives on diagnostic device construction are described.

## 2. Isothermal Nucleic Acid Amplification

Isothermal approaches can facilitate rapid target amplification through single-temperature incubation, reducing system complexity compared to PCR-based methods. Established isothermal amplification methods differ in terms of complexity (multiple enzymes or primers), attainable sensitivity, and specificity. In this section, we introduce the main isothermal methods used in diagnostic systems, including nucleic acid sequence-based amplification, strand displacement amplification and rolling circle-based, loop-mediated, and helicase-dependent amplification. In addition, we discuss the potential of new isothermal methods such as beacon-assisted detection amplification and hybridization chain reaction.

### 2.1. Nucleic Acid Sequence-Based Amplification

**Figure 1.**
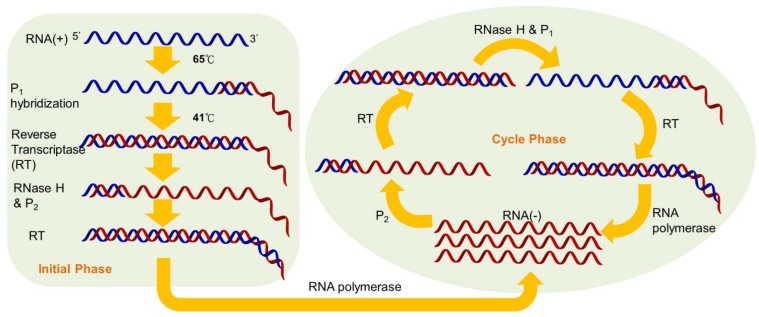
Schematic representation of the NASBA amplification.

Nucleic acid sequence-based amplification (NASBA) is a sensitive transcription-based isothermal amplification system for mimicking retroviral RNA replication. The single-stranded RNA product is a desirable target owing to the integration of reverse transcriptase into the amplification reaction. Thus, the NASBA assay has been widely employed for identifying bacterial and viral RNA in clinical samples [6–9]. The reaction is performed with a two-stage protocol: the initial phase for denaturation and primer annealing at 65 °C, and the cycle phase for target amplification at the predefined temperature of 41 °C. At the initial phase, the reverse primers (P_1_), which contain polymerase promoter sequences, are extended by the activity of reverse transcriptase, after which the RNA of the RNA-cDNA hybrids is degraded by RNase H (Figure 1).

The forward primers (P_2_) hybridize with the cDNA strands to form new templates that are suitable for the extension of reverse transcriptase. The double-stranded promoter sequences can again be produced, and simultaneously, newly synthesized RNA fragments will be formed by the polymerase, thus initiating the next round of the cyclic phase [10]. In addition to offering an isothermal and convenient process, the major advantage of NASBA compared with RT-PCR is the production of single-stranded RNA products, leading to simple detection using probe hybridization without any denaturation step. Additionally, NASBA has been shown to have sensitivity comparable to that of RT-PCR [11–13].

To monitor the amplified target during the NASBA process, molecular beacon probes are designed to hybridize with the product, generating a specific fluorescent signal. With an increasing number of NASBA cycles, more and more amplified RNA sequences are produced and then interacted with the molecular beacon probes during the annealing stage. This sensing strategy generates increasing signals from the molecular beacon, and the fluorescence provides a real-time monitoring of NASBA progress [5,14,15]. Recent effort has shown that an automated NASBA system, NucliSENS EasyQ, can perform simultaneous amplification and detection using fluorescence quantification. The detection of amplification products takes place in a single closed tube to significantly reduce contamination risks. This platform also helps decrease the hands-on time and provides rapid results (within 4 h), thus becoming a potentially suitable device for diagnostic applications [16–19].

Although the NucliSENS EasyQ platform can obtain measurements simply and rapidly at central laboratories, the system has had limited application outside of this context. With the goal of bedside monitoring, many researchers have reported on integrated analysis systems that make it possible to shift NASBA applications from high-cost, tabletop systems to low-cost, portable devices. Esch *et al.* developed a NASBA assay in conjunction with fluorescence detection on a microfluidic device [20]. This device consisted of a polydimethylsiloxane (PDMS) block with a single channel, placed on a gold-coated glass slide at the device's center to immobilize the probe. Detection was accomplished using a sandwich hybridization of the NASBA products between capture probes and reporter probes tagged with carboxyfluorescein-filled liposomes. This technique had a detection limit of 5°fmol/L for a sample size of 12.5 μL. A later publication by Dimov *et al.* reported a microfluidic diagnostic device that integrated solid-phase extraction, real-time fluorescence detection, and a NASBA assay [21]. The integrated microfluidic NASBA chip consisted of two reaction chambers: a silica bead-bed chamber for sample purification and concentration, and a NASBA chamber for RNA amplification. To improve the efficiency of the NASBA reaction, all chambers were incubated with bovine serum albumin overnight before the reaction was started. Adequate amounts of the NASBA product were obtained after a reaction time of 30 min. Earlier this year, Zhao *et al.* introduced the concept of an integrated microfluidic chip-based system to monitor pathogens in a water environment with femtomolar sensitivity. The system, called immuno-NASBA, combined the versatility of enzyme-linked immunosorbent assay (ELISA) with the amplification power of NASBA [22]. The device was modeled on a 96-well ELISA microplate with 43 reaction chambers so that it would be fully compatible with a conventional reader. Moreover, the chip contained six parallel reaction channels to perform the simultaneous detection of six targets. Immuno-NASBA diagnostic devices have powerful potential to be applied for the diagnosis of various infectious diseases.

### 2.2. Strand Displacement Amplification

**Figure 2.**
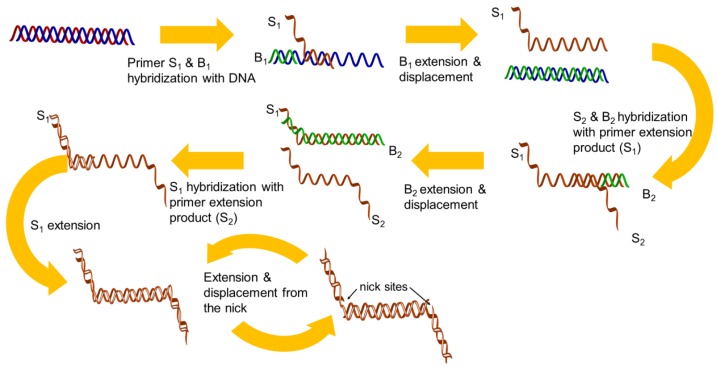
Schematic representation of the SDA reaction.

Strand displacement amplification (SDA) was described in 1992 [23] and was improved in the same year [24]. There are four sequence-specific primers used in this isothermal amplification. The first set of primers (S_1_ and S_2_) is designed to have single-stranded restriction enzyme recognition site overhangs, and the second set of the primers (B_1_ and B_2_) represent the bumper primers. The DNA target is first denatured by heat and each strand is allowed to hybridize with two primers (S_1_ and B_1_), which are annealed to the DNA template. The B_1_ extended product displaces the extension from the S_1_ primer, which can hybridize to the opposite strand primers (B_2_ and S_2_). Thus, newly synthesized DNA that has been extended from the primers is cleaved by a corresponding restriction endonuclease, and the amplification is repeated by the polymerase, thus generating the newly synthesized strands (Figure 2). Unfortunately, the technique still requires a higher temperature (95 °C) to perform a denaturation step for initial primer binding and amplification.

BD ProbeTec ET is a semiautomated real-time system for carrying out SDA with fluorescence detection, which has proven useful in the detection of bacteria in clinical samples [25–31]. The BD ProbeTec ET system offers a number of advantages over traditional culture-based methods, especially in the detection of Chlamydia trachomatis and Neisseria gonorrhoeae. The operation of this detection platform is no more technically difficult than conventional approaches. Total processing time for bacteria detection using this system takes less than 5 h from specimen receipt, as opposed to 4–8 weeks for the completion of traditional cultures. Such a rapid turnaround time combined with excellent device characteristics and ease of use can considerably improve patient care. In addition, a more rapid diagnosis of infected patients will facilitate the maximum possibility of limiting dissemination. Briefly, this diagnosis device offers a more rapid and cost-effective way for screening the spread of infectious bacteria.

In another design, He *et al.* incorporated SDA to a lateral flow strip in an effort toward more rapid diagnostics [32]. Nucleic acids are captured on strips in an antibody-dependent manner, using an antibody capture line and an oligonucleotide probe of complementary sequence to the amplicon. The developed SDA strip allows the detection of keratin 10 in epidermolytic hyperkeratosis produced at 1 fm within 75 min.

### 2.3. Rolling Circle Amplification

**Figure 3.**
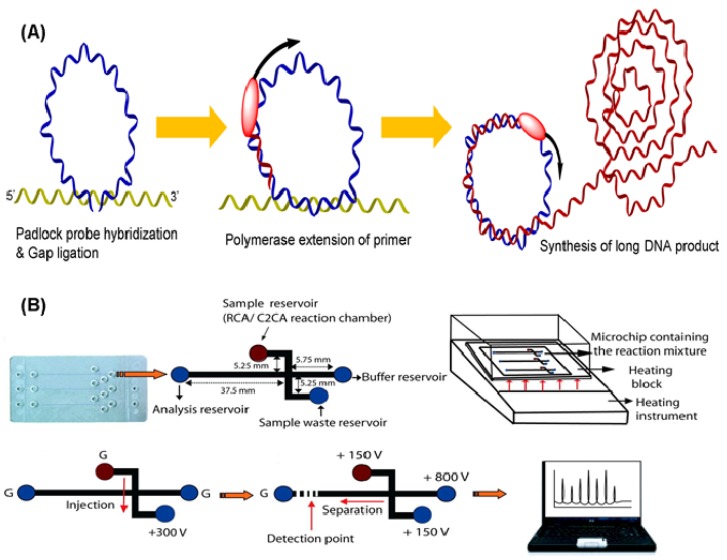
(**A**) Schematic illustration of the principal steps of RCA. (**B**) System design for on-chip RCA integrated platform (reprinted with permission from [60]. Copyright 2008 American Chemical Society.)

Rolling circle amplification (RCA) is a representative amplification technique that uses a single DNA primer and padlock probes. The probes, including two target-complementary portions linked by a connecting sequence, are designed to circularize via ligation with the DNA target strand [33]. In the beginning, the end portions of the probe hybridize two consecutive sequences in the target sequence, thus constructing a circle with a nick that is closed by ligation. After ligation, circular padlock probes used as templates can be copied by DNA polymerases from phage φ29, resulting in multiple copies of the circle DNA sequences (Figure 3(A)) [34]. This polymerase, with an excellent strand displacement activity is important for efficient RCA because it can continuously progress around the circle probe and displace the amplified fragment, generating a long ssDNA product. The technique provides several advantages such as simplicity and robustness over other isothermal amplification technologies. Over the years, different circle amplification assays have been developed for molecular monitoring by using single-primer initiated RCA, two-primer amplification [35], multiply-primed strategy [36], and dubbed-primer generation [37]. Because the operation of RCA is at moderate temperature and pH, this assay does not affect antigen-antibody binding [38]. Consequently, it has been extensively employed in DNA [39–41] and protein [42–44] detection.

To date, various sensors have been developed with RCA based on electrochemistry [45–47], optics [48–50], fluorescence [51–53], chemiluminescence [54,55], and other assays [56]. Schopf *et al.* presented a bead-based RCA in a sandwich assay to detect viral DNA by hybridizing padlock probes with 1 μm diameter beads as surfaces for RCA [57]. Because of the immobilization of RCA products on beads, this on-chip RCA can decrease complexity and increase fidelity. Sato *et al.* created a microchip-based on-bead RCA system that integrates all operating processes into a chip for rapid nucleic acid quantification [58]. A glass microchip consists of a Y-shaped microchannel with a dam structure, which is used to localize beads during RCA steps. In addition to DNA detection, Yan *et al.* reported the first example of protein detection by on-nanoparticle RCA [59]. Magnetic particles were introduced to separate the target from body fluid. Baba's group has reported an integrated poly(methyl methacrylate) (PMMA) microchip using an electrophoretic port as an RCA reaction chamber for rolling-circle and circle-to-circle amplification and the subsequent microchip electrophoretic analysis of bacterial genes (Figure 3(B)) [60,61]. A clinical sample was detectable in less than 65 min after the reaction was initiated.

In addition to single-target detection, RCA is also desirable for multiple-analyte sensing assays because amplified products are considered to be localized at the array spot [62]. An array of real-time RCA in combination with the parallelism of arrays was developed by Yang *et al.* for protein quantitation down to the low nanomolar range [53]. Konry *et al.* constructed a two-layer sandwich assay on microbead surfaces for the combined detection of DNA and protein molecules in a single approach [63]. This array chip achieved detection limits of 1 pM and 10 fM for target DNA and proteins, respectively.

### 2.4. Loop-Mediated Isothermal Amplification

**Figure 4.**
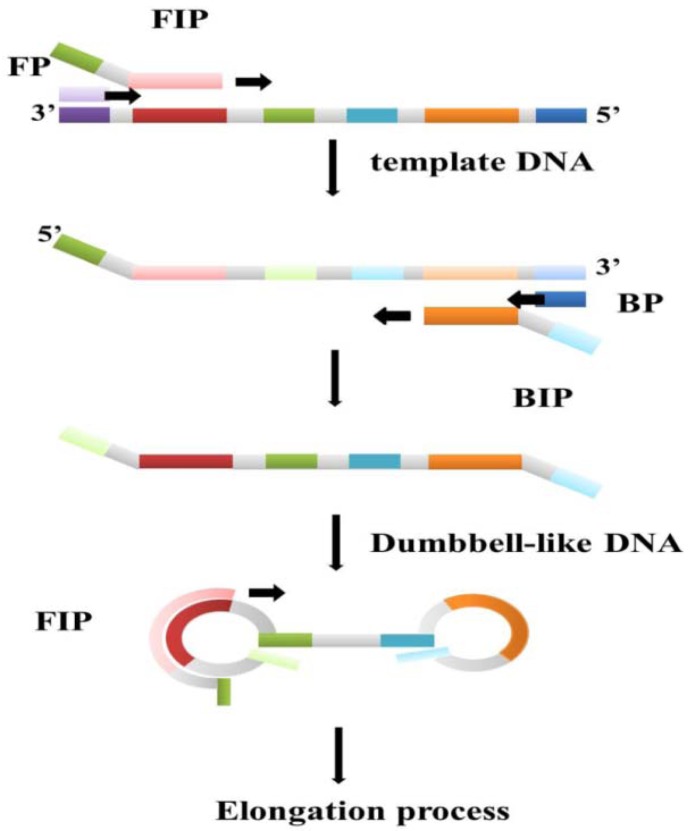
Schematic illustration of the principal steps of LAMP.

Loop-mediated isothermal amplification (LAMP) is one of the DNA amplification technologies that employ a constant temperature [64]. The Bst polymerase plays a key role in the LAMP reaction process. The Bst polymerase, which is derived from *Bacillus stearothermophilus* living in hot springs with temperature around 70 °C, has polymerize activity, 5′-3′ exonuclease activity, and strand displacement ability. At a suitable temperature, Bst polymerase with strand displacement activity can separate the non-template strand from the template DNA without the thermal cycles of the PCR process, which uses Taq polymerase to synthesize new DNA strands. Subtle primer design is also necessary for a successful LAMP reaction. In the first stage of the reaction, the so-called outer and inner primer pairs can make dumbbell-like loop DNA strands from the target DNA templates, and the dumbbell-like DNA strands become the new template DNA for the next step (Figure 4). The dumbbell-like DNA strands then continue replicating to become a flower-like long-chain DNA product [65]. In addition to these two primer pairs, a third pair known as loop primers has been designed and proven to be beneficial in accelerating the amplification process. A good primer design not only ensures successful execution of LAMP, but also increases the sensitivity and specificity of the reaction result [66]. Thus, the LAMP reaction is carried out by three pairs of primers in an isothermal condition. Compared to the PCR, the reaction time of LAMP is shorter while the sensitivity and specificity are almost the same or even better. For fixed temperature heating, the heater component of the device can be simpler relative to traditional DNA amplification instruments. These features afford LAMP strong potential as a disease screening method based on the economic benefits of clinical point-of-care devices with simpler designs. Because of convenience, high efficiency, and the specificity of LAMP, it has been applied to many DNA screening tests, especially virus detection.

Microfluidic chips have been applied to the detection of LAMP reactions in recent years. Some chips are used only for guiding the reaction buffer and DNA solution to the reaction chamber, whereas others are combined with additional technologies such as nanostructures for sample concentrating, electrophoresis, magnetics beads, *etc.* A microfluidics chip made of PMMA has been used for the turbidity detection of the hepatitis B virus (HBV) LAMP reaction by our group [67,68]. With a disposable LAMP microreactor and optical fiber-based turbidimetry device, as shown in Figure 4, the lowest limitation for detection of the HBV DNA template was 50 copies/25 μL with the critical detecting time set at 30 min.

A multichannel microfluidics device for LAMP based on turbidity detection was developed by Fang *et al.* [69]. In this research, pseudorabies virus, the swine virus, was used as the test model, and the device needed only a small sample volume of around 0.4 μL for a complete time of less than 1 h. Fang *et al.* also demonstrated another multiplex microfluidics LAMP device that distinguished three types of human influenza A substrains (fluA, seasonal H1N1, and pandemic H1N1) with a sample volume of only 2 μL and a detection limit of less than 10 copies/μL [70]. A further design of microfluidics with microvalves and micropumps improved the controllability of the reagent adding process in the LAMP reaction. A thermoresponsive polymer microvalve was used in a LAMP microchannel device to build a closed reaction chamber that prevents the evaporation or outflow of fluids during the heating process [71]. The valve was made using PDMS with an expandable microsphere filled with isobutene and isopentane and a shell of vinylidene chloride, acrylonitrile, and methyl methacrylate. After heating to 80 °C, the microsphere expands and the valve containing the sphere beads closes the microchannel to leave the buffer injected inside the reaction chamber. The valve can withstand a maximum of 200 kPa, so that it can tolerate the internal pressure produced by heating. In addition to fluid control, sample preparation is also an important step that can be integrated into the microfluidics system. Solid phase extraction micropillars made of glass have been combined with a LAMP microfluidics chip for nucleic acid extraction [72]. Nucleic acids tend to adhere to the glass pillars in a chaotropic salt guanidine hydrochloride environment. After the extraction of DNA, the sample can be eluted by a low ionic strength solution to the LAMP reaction chamber for amplification. With the exception of unibody micropillars, additional extraction components have also been used in the LAMP device. Liu *et al.* used the Flinders Technology Associates (Whatman FTA^®^) membrane embedded to the channel to extract the DNA, and the LAMP reaction was performed right on the membrane without any elution process [71]. Hence, based on the extraction of nucleic acid and the removal of the LAMP inhibitor in saliva, the detection limit of HIV DNA was improved to 10 copies in a reaction time of less than 40 min by adding DNA inside the reaction chamber.

**Figure 5.**
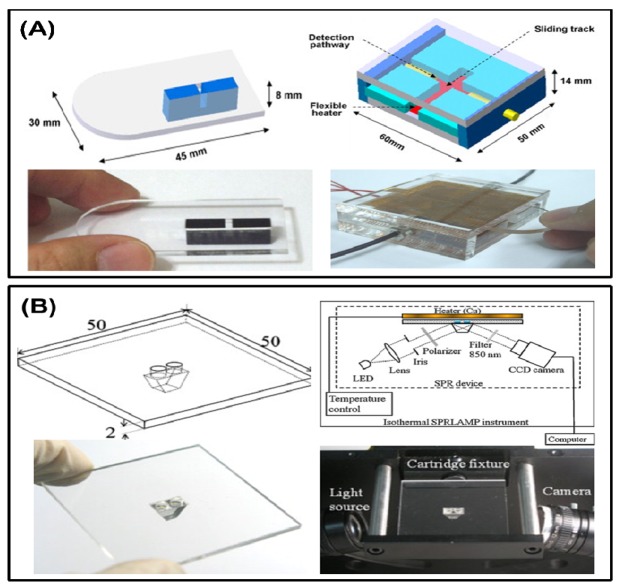
(**A**) Schematic diagram of an integrated LAMP turbidimetry device. (Reprinted with permission from [68]. Copyright 2007 Elsevier B.V.) (**B**) Schematic diagram of an SPR-based LAMP sensing cartridge and system (reprinted with permission from [74]. Copyright 2012 Elsevier B.V.).

Besides fluorescence, which is commonly used in DNA detection, the turbidity change from magnesium sulfide production during LAMP reaction is also an important character. A single-channel turbidimetry instrument was assembled to a small device by Lee *et al.* as previously mentioned (Figure 5(A)) [67,68], and in another device, the parallel measurement of the LAMP reaction was performed by a commercial portable turbidimeter [73]. The detection limitation can be lowered to 100 fg total RNA for the Taura syndrome virus, as demonstrated by Sappat. Another high sensitivity and fast surface plasmon resonance (SPR) platform for the LAMP reaction is reported by Chuang *et al.* [74]. The SPR setup consists of an 850 nm light-emitting diode (LED), a charge-coupled device (CCD) camera, and a disposable polycarbonate-based reaction chamber with a prism (Figure 5(B)). Instead of sensing the DNA directly, the research report revealed a new strategy to measuring the change in the refraction index of the bulk solution during the reaction. The results showed that a detection limitation at 2 fg DNA per microliter within 20 min was achieved.

Electrochemistry has been considered a possible method for the miniaturization of the optical detection component of a microfluidics system [75–78]. Nagatani *et al.* used a universal serial bus (USB) power-controlled electrode for the RT-LAMP detection of the influenza virus and yielded the peak height in the I-V curve for analysis [78]. The threshold for the calibration curve was determined using serial diluted positive control. The viral load of the influenza virus was estimated by the area under the curve.

### 2.5. Helicase-Dependent Amplification

**Figure 6.**
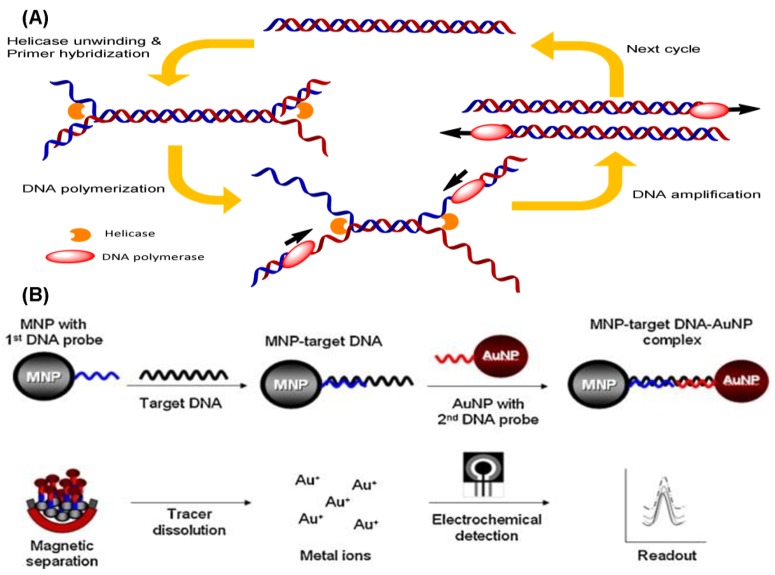
(**A**) Schematic illustration of the principal steps of HDA; (**B**) System design for an on-chip RCA integrated platform (reprinted with permission from [82]. Copyright 2011 Elsevier B.V.).

Helicase-dependent amplification (HDA) is based on natural DNA replication mechanisms. Initially, the coordinated action of helicases unwinds and separates the template DNA duplex. The primer can hybridize with the free single-stranded templates, and the subsequent extension by a DNA polymerase will result in DNA amplification (Figure 6(A)). The original reaction reported in the literature is performed at 37 °C for the entire process, and more than a million-fold amplification of DNA fragments can be achieved from nanogram quantities of genomic DNA [79]. Unlike the PCR, HDA uses helicases instead of heat, thus eliminating the need for any denaturation steps. Nevertheless, two additional accessory proteins are required in this approach: MutL to stimulate helicase unwinding activity and a single-strand binding (SSB) protein to prevent premature re-association of the separated ssDNA. A thermostable helicase may be also advantageous for HDA. Recently, a new helicase was developed from *Thermoanaerobacter tengcongensis*, which can be operated at temperatures from 45 °C to 65 °C [80], so HDA reactions are now generally performed at the higher temperature of 65 °C. The use of thermostable helicase led researchers to abandon both the MutL and SSB proteins, while simultaneously improving the DNA yield of the reaction [81]. This simple thermal management option makes HDA very attractive for the development of simple portable DNA diagnostic devices and point-of-care testing.

Recently, electrochemical methods for the detection of DNA in combination with HDA have been developed. A DNA-based sensor for the detection of *M. tuberculosis* using the electrochemical detection of gold nanoparticles was developed [82]. The dextrin-coated gold nanoparticles (AuNPs) used as a reporter can be electrochemically detected on a screen-printed carbon electrode chip (Figure 6(B)). Kivlehan *et al.* developed a real-time electrochemical method for HDA using the monitoring of intercalating redox probes [83]. The binding of redox probes to the HDA products (amplified double-stranded DNA) led to less electrochemically detectability, compared with the probes' free counterpart. This method of electrochemical HDA detection does not require the immobilization of the probe on the electrode; real-time isothermal HDA reactions with 48-electrochemical microwells can be performed in 1 h. Therefore, it has the potential to be a reliable method for sequence-specific DNA detection.

Lateral flow test strips provide a promising tool for the development of point-of-care nucleic acid biosensors. Consequently, HDA has been employed with an embedded lateral-flow DNA detection strip for end-point assay to detect HIV-1 in human plasma [84]. The principle of this approach is based on a sandwich immunoassay using two probes: a fluorescein isothiocyanate (FITC)-labeled capture probe and a biotin-labeled detection probe. The HDA products hybridize with the capture probes and detection probes to form the complex. The hybrids are bound to streptavidin-conjugated color particles and are captured on the test zone by the interaction between the target DNA-FITC capture probe and an anti-FITC antibody. The accumulation of color beads in the test zone of the fiberglass paper is visualized as a characteristic red band. This assay provides the satisfactory detection of HIV-1 RNA at 50 copies/assay. This disposable amplicon detection device based on HDA has also been applied to the herpes simplex virus [85] and *Mycobacterium tuberculosis* diagnosis [86] and shows a performance comparable with conventional detection assay. Nevertheless, sample preparation, target amplification, and nucleic acid testing are conducted as distinct steps.

Andresen *et al.* performed HDA in a microarray format, making it suitable for the simultaneous detection of various pathogens [87]. In their reported technique, the first primer was immobilized onto the microarray surface and then, the second primer, labeled with a reporter, and enzymes were mixed in the HDA reaction sample. In the absence of pathogens, no labeled amplification product was detected. The detection of the two pathogens *N. gonorrhoeae* and *S. aureus* in single- and duplex-format of OnChip HDA was carried out within 2 h. This study demonstrated that HDA with a chip-based array can provide unambiguous visible results. Microfluidic DNA assays involving on-chip isothermal amplification have also been developed for HDA. Ramalingam *et al.* reported a microchip-based HDA system for the amplification of a 78 bp fragment of severe acute respiratory syndrome (SARS) complementary DNA [88]. The diagnostic device, with an unsealed PDMS/glass-multiplexed microreactor, contained four parallel 5 μL microchambers. The methods of fabrication used in this study are simple, and the operation of the device is straightforward, requiring no pumps or valves, only capillary action for liquid handling. An absorbent pad was placed at the end of the sample loading channel to remove excess amplification mixture. The HDA primer pairs were patterned onto the glass substrate during fabrication and dried before bonding to the molded PDMS reactor. Thus, the dried primer pair was inside each microchamber, which can be an interesting microfabrication procedure. These advantages and features help make these devices applicable for the real-time detection of SARS cDNA with a detection limit of 0.1 ng/μL. Additionally, this device used small reaction volumes (192 nL–5 μL), which can potentially lead to short amplification times. Although this microchip-based reactor included integration with on-chip sample amplification and isolation, the purification step was not performed on-line with HDA. Recently, Mahalanabis *et al.* reported an integrated system that performed DNA purification from whole bacteria in a liquid sample, followed by HDA and nucleic acid hybridization [89]. This system allowed multiple operations to be performed on chip, and thus, the total detection time including sample preparation was 50 min. Such an integrated microfluidic device, with its ease of use and short detection time, can become a portable device for rapid, label-free, specific detection.

### 2.6. Other Isothermal Amplification of Interest

Trau's group has proposed a beacon-assisted detection amplification (BAD-AMP) by DNA polymerization in conjunction with the nicking event [90,91]. Two enzymes are used in BAD-AMP: the DNA polymerase that replicates the DNA target on the beacon and the nicking endonuclease that cuts the replicated single strand at the recognition position. Initially, the reaction can be activated by the addition of target DNA to switch the conformation of the beacon. When a new DNA is synthesized, the target is displaced by the polymerase with strand-displacement activity. This polymerization eventually leads to the newly synthesized DNA strand with a recognition sequence for the DNA endonuclease. This allows an enzyme to nick the DNA strand, such that the polymerase can also displace the nicked strand. BAD-AMP leads to exponential amplification by repeating cycles of polymerase and endonuclease activity. Because this strategy is a relatively simple technique, BAD-AMP has also been applied for the construction of molecular logic gates [92].

Hybridization chain reaction (HCR) is a short DNA amplification technique that is based on hybridization and strand-exchange reactions for selective and specific extension [93]. Two complementary, kinetically trapped DNA hairpins coexist in solution until the introduction of target strands initiates a cascade of hybridization events. Because there is no requirement for enzyme amplification of the signal, HCR can be performed at room temperature. The major drawback of HCR is that it provides linear amplification only, compared to the PCR, which produces exponential amplification. Various approaches with labeled hairpin probes have been reported to improve the sensitivity of targets [94–97]. Although HCR is the simplest method among the isothermal nucleic acid amplifications, there are no reports on the development of an integrated HCR chip.

## 3. Conclusions

The aim of this review was to briefly describe the current state of the art of diagnostic devices for isothermal nucleic acid amplification. The isothermal strategy has been a versatile and powerful technique applied in the detection of microbial and viral pathogens, among many other uses in the diagnostic laboratory. The combination of the properties derived from isothermal amplification and biosensing platforms proved a valuable strategy for simplifying the analytical science of nucleic acid detection. In reviewing the various detection configurations, we observed that integrated microchip systems are particularly desirable because these systems provide significant advantages in convenience and cost-effectiveness, simultaneously simplifying operational procedures and shortening analysis times.

To date, the development of chip-based isothermal assay systems has received great attention, whereas achieving a higher degree of portability remains a challenge. No device reported thus far is clearly superior, resulting in the possibility that sensing platforms based on different isothermal amplifications may find their way to market. Commercialization requires further improvement in on-chip sample pretreatment, miniaturization of detectors, decrease in power consumption, and the establishment of quality control. We can expect the full integration of all components on disposable credit-card-sized systems for isothermal nucleic acid amplification and detection in the near future. Given the great effort being invested in isothermal DNA microchip systems, there is no doubt that they will provide significant contributions to point-of-care diagnostics and decentralized testing.
